# Investigations and Outcomes for Olfactory Disorders

**DOI:** 10.1007/s40136-022-00438-x

**Published:** 2022-11-29

**Authors:** Louis Luke, Liam Lee, Lavandan Jegatheeswaran, Carl Philpott

**Affiliations:** 1 Ear, Nose and Throat (ENT) Department, James Paget University Hospital, James Paget University Hospitals NHS Foundation Trust, Great Yarmouth, UK; 2 Norwich Medical School, University of East Anglia, Norwich, UK

**Keywords:** Olfaction, Olfactory disorders, Anosmia, Hyposmia, Investigation, Outcome

## Abstract

**Purpose of Review:**

To provide a detailed overview of the investigations and core outcome measures for olfactory disorders.

**Recent Findings:**

Olfactory disorders can have a detrimental impact to the quality of life of patients. There are a wide range of causes of olfactory loss including sinonasal conditions, idiopathic, post-head trauma or infection. This review highlights the key investigations and reasoning for their use to clinically assess and research patients with olfactory disorders. In addition, this review outlines the core outcome measures for olfaction that will help inform future research in olfactory disorders.

**Summary:**

A systematic approach with history taking and examination particularly with nasal endoscopy can determine the cause of the olfactory disorder in most cases. Specific olfactory disorder questionnaires can demonstrate the impact on quality of life, while psychophysical testing can objectively assess and monitor olfaction over time. Olfactory-evoked potentials and functional MRI are reserved for research, whereas CT and MRI imaging are used depending on history and examination. A core outcome set for olfaction has been developed that will help standardise the outcome measures used in olfaction and olfactory disorders research.

## Introduction

Olfaction is an essential sense for daily life. The sense of smell impacts a person’s quality of life in several ways. It provides information about potential hazards in the environment such as smoke, gas, or spoiled food. It also influences the perception of pleasure through food, sexual relationships, and mood [1]. Olfactory loss is a relatively common condition with a prevalence of 2.7 to 24.5% from population-based epidemiological studies [2]. It is well recognised that olfactory loss can have a significant detrimental impact to the wellbeing of patients including the negative effects on emotional states, relationships, and physical health [3, 4]. Unfortunately, olfactory disorders are not given the same emphasis in the medical profession as auditory or visual impairment, and many patients report negative encounters with clinicians as a result of this [5].

Olfactory disorders can be categorised into quantitative and qualitative smell disorders. Quantitative smell disorders include anosmia (complete smell loss) and hyposmia (reduction in the sense of smell). Conversely, qualitative smell disorders include parosmia (distorted perception of smell in the presence of an odour source) and phantosmia (odour perception in the absence of stimuli) [2, 4]. Olfactory disorders can be categorised as conductive (obstruction of the transfer of odorant to the olfactory neuroepithelium), sensory (olfactory receptor dysfunction), or neural (dysfunction of central olfactory pathways) causes. Conductive causes typically include sinonasal disorders such as chronic rhinosinusitis, foreign bodies, or tumours whereas post-infectious olfactory dysfunction (PIOD), post-traumatic olfactory dysfunction (PTOD), Alzheimer’s disease, and Parkinson’s disease are typical examples of sensorineural causes. Some causes may affect the olfactory pathways in more than one way such as CRS and toxic rhinitis, with conductive and sensory elements possible. Dam et al. identified the following frequencies for causes of olfactory dysfunction, sinonasal conditions (67%), PIOD (14%), idiopathic (8%), PTOD (6%), and other causes including iatrogenic, congenital, and toxic (5%) [6]. Recently, PIOD has become increasingly prevalent due to the coronavirus (COVID-19) pandemic in 2020. It is well recognised that olfactory loss was a hallmark symptom of COVID-19 infection that can be transient in the majority of patients but can also result in permanent olfactory dysfunction [7, 8]. Given the range of underlying causes and their impact, it is important to undertake a thorough clinical assessment for olfactory dysfunction to help guide management and improve the quality of life of these patients.

This article reviews the investigations for patients presenting with olfactory dysfunction explaining the reasoning behind their use in addition to reviewing the core outcome measures that should be used to assess olfaction for clinical and research purposes.

## Investigations for Olfactory Disorders

### History

A systematic approach is required in assessing a patient with olfactory dysfunction. The international consensus statement on allergy and rhinology: olfaction (ICAR:O) strongly recommends a complete olfaction history and examination is undertaken. There is emphasis on the importance to enquire about the quality of olfactory dysfunction such as anosmia, hyposmia, phantosmia, or parosmia as described above to determine a qualitative or quantitative cause. Furthermore, questions on laterality, the patient’s perceived degree of smell loss, their sense of smell before loss, timing of onset, duration, and whether symptoms are persistent or intermittent can help a clinician understand a patient’s olfactory loss [9••]. Perceived gustatory loss is a common complaint associated with olfactory dysfunction due to olfaction comprising a large component of flavour perception. However, true gustatory loss is rare but and thus the taste sensations of sweet, salt, sour, bitter, and umami are typically preserved in the majority of patients [10, 11]. As mentioned earlier, there are various causes of olfactory disorders and therefore enquiring about factors associated with these are important such as sinonasal symptoms, preceding viral infections, head trauma, surgery, or malignancy. Other important factors to elicit include medication, mental health or neurodegenerative disorders, and social history, particularly smoking and occupational exposure [12, 13].

### Examination

Nasal examination is fundamental to investigate olfactory disorders. Anterior rhinoscopy can help identify anterior deformities causing a conductive loss of olfaction including septal deviation and inferior turbinate enlargement. Flexible or rigid endoscopy ensures a thorough assessment of the nasal cavity and nasopharynx to identify signs of inflammation, discharge, polyps, crusting, scarring, or masses particularly at the olfactory cleft and ostiomeatal complex [9••, 13]. Seiden and Duncan stressed the importance of performing endoscopic examination to discern the presence of conductive olfactory disorders as anterior rhinoscopy alone failed to identify diagnose pathology in 51% of cases compared to 9% failure with nasal endoscopy [14]. Endoscopic findings can be objectively measured and documented using validated scoring systems including the Lund-Kennedy scoring system for chronic rhinosinusitis or olfactory cleft endoscopy scale [15, 16]. Intranasal anaesthesia for endoscopy should be judiciously used or used after history taking and chemosensory testing due to its potential of temporary anosmia and reduced perceived self-assessment of olfaction [17]. Further examinations should be guided by the history or nasal examination findings. For example, a full peripheral nerve and cranial nerve examination should be undertaken if there is a suspicion of an underlying neurological cause. Likewise, a head and neck examination will be required for medico-legal purposes or where there are suspicions of trauma or malignancy from the history or nasal examination [18].

### Subjective Assessment of Olfaction

Subjective assessment includes the use of visual analogue scores to gauge self-perception; however, there is a recognised lack of correlation between self-reported olfaction related visual analogue scales and psychophysical test results [19, 20]. Self-reported questionnaires such as the Questionnaire of Olfactory Disorders Negative Statements (QOD-NS) have been shown to be able to differentiate between normosmia and hyposmia but have the added benefit of assessing qualitative disorders such as phantosmia and parosmia [21, 22]. Langstaff et al. demonstrated no correlation between the English Olfactory Disorders Questionnaire (eODQ) scores and Sniffin Sticks psychophysical test scores emphasising the importance of assessing the impact of olfactory disorders on quality of life in the clinical setting [23]. Therefore, it is recommended that subjective assessment should be used in combination with psychophysical testing to provide an overall assessment of olfactory function [18].

### Psychophysical Testing of Olfaction

Psychophysical olfactory testing is performed to quantitatively measure olfactory function and confirm the presence of olfactory dysfunction. It also enables the monitoring of changes in olfactory function over time and may help to detect malingering behaviour in medico-legal cases [10, 24]. Odours are presented to a subject, and their responses are monitored with scores adjusted for age and sex [25]. Odours can be presented either through orthonasal olfaction, where the odour is sniffed through the nostrils or by retronasal olfaction, where the test odours enter through the nasopharynx through the use of powders [26–29]. There are three common modalities of psychophysical assessment: odour threshold, odour discrimination, and odour identification testing. For odour threshold testing, different concentrations of a particular odorant (n-butanol or 2-phenylethyl alcohol) are presented to a subject in an ascending manner until the lowest concentration that the subject perceives is detected. This mainly assesses peripheral olfactory receptor sensitivity. Suprathreshold (high concentration) typically testing assesses central olfactory function by odour discrimination, the subject detects the abnormal odour from a series of odours and odour identification, and the subject identifies the exact odour from their semantic memory [30]. It is important to note the influence of culture on odour identification tests as some odours may be unfamiliar to subjects of different cultural backgrounds. Therefore, a psychophysical test should be appropriately adapted for the subject population studied [25]. There are a wide variety of validated psychophysical olfactory tests available, but it is important to use a culturally adapted one. For example, the descriptors of the identification part of the Sniffin’ Sticks test were adapted in a British population to be more culturally familiar than their German originators [38]; these modified descriptors demonstrated an increase in reliability of the SS when used for a British population [39]. Fahmy and Whitcroft have conducted a detailed review of psychophysical testing used for chemosensory disorders [31•]. Below is an overview of different psychophysical testing methods.

There are a number of orthonasal psychophysical tests that present odours in a variety of ways such as encapsulated odours, pens, jars, or filter paper [32–35]. Forced-choice odour identification testing (a suprathreshold odour is presented to a subject whom must identify the odour from a list of descriptors) is preferred compared to other test methods due to its increased reliability, correlation with other tests, and being pragmatic in the clinical and research setting [9••]. As previously mentioned, increased test length corresponds with increased reliability and thus validity of a test [36]. Therefore, it is recommended that short screening tests should be used to identify subjects with olfactory dysfunction, whilst longer tests are used to quantitatively assess the degree of olfactory dysfunction [9••]. The most widely used and validated psychophysical tests are the Sniffin Sticks (SS), the University of Pennsylvania Smell Identification Test (UPSIT), and the Connecticut Chemosensory Clinical Research Center (CCCRC) test [18, 30].

Another method of psychophysical testing is retronasal testing where odours are presented to the posterior part of the nasal cavity through the oral cavity. The reason for performing retronasal testing is when there is a perceived mismatch between orthonasal and retronasal olfaction that is not accounted for by any gustatory component. As the flavour of food relies on both orthonasal and retronasal olfaction, a food that may have a foul smell may have a pleasant taste [27]. There are numerous methods of retronasal testing. The most used technique is the retronasal olfaction test (ROT) which was first introduced by Heilmann et al. [28]. Twenty food powders are presented onto the tongue using squeezable plastic vials, whilst the subject’s nose is clipped. A forced-choice odour identification test method is used with 4 possible options and responses recorded. This test showed a high test–retest reliability correlation (*r*_27_ = 0.76) in healthy subjects that was similar with SS TDI scores as well as the ability to discriminate between anosmic, hyposmic, and normosmic subjects [28]. A systematic review highlighted the lack of knowledge on retronasal olfaction thresholds and thus the optimal concentrations and appropriate test odours [37]. In terms of investigating olfactory disorders, retronasal testing should be selected in the clinical setting where its evaluation may be beneficial such as in patients complaining of associated gustatory disturbance [29].

As mentioned earlier, psychophysical testing has traditionally been limited to quantitative measurement of olfactory dysfunction. The Sniffin’ Sticks Parosmia Test (SSParoT) using the SS is a recent attempt to measure qualitative olfactory dysfunction by assessing the hedonic range. This test determines the perceived hedonic distance between two opposing odours and hedonic direction, as well as the overall hedonic perception of odours [38].

### Olfactory-Evoked Potentials

Measurement of olfactory-evoked potentials is an objective approach to assessing the neuronal activity of olfaction through the detection of brain or electrical activity of the olfactory pathway in response to stimuli [39]. Different methods have been developed to capture such data.

Electroencephalography (EEG) measures electrical waves due to synchronous neuronal activity in the brain by attaching probes to the scalp of the subject [40]. Olfactory event-related potentials (OERPs) and olfactory chemosensory event-related potential (CSERP) are both extensions of EEG. The difference between OERP and CSERP is to distinguish the pure olfactory perception from olfactory perception with trigeminal nerve stimulation. OERP therefore uses pure odorants (2-phenylethyl alcohol, vanillin), while CSERP uses stimuli that can stimulate the trigeminal nerve (carbon dioxide, pheromones) [41]. Regardless of the type of stimulants used, both methods allow detection of changes in brain wave frequencies. The presence of OERP activity was estimated to be significant at a TDI score of 22.6 [42]. Therefore, OERP activity may be useful to confirm the presence of olfactory function, but its absence does not necessarily confirm the absence of olfactory function. Despite there being clear changes in brain wave frequencies when a subject is exposed to odorants, the precise characteristics of changes are not consistent. For instance, different waveforms like α wave and θ wave have been suggested to be the primary statistically significant variable when a subject is exposed to different odours [43]. EEGs are difficult to interpret, but there are also technical challenges such as the requirement for stimuli to be delivered in a highly controlled manner. Brain activity varies depending on concentration, flow rate, temperature, and humidity of the airstream containing the odour [44]. To mitigate this issue, olfactometers were developed to facilitate this requirement for tight regulation in many parameters [45]. Despite great progress in technology, the application of OERP and CSERP is limited in normal clinical settings, and these tests are not routinely used outside of research purposes, although electrophysical testing can be useful in the medico-legal setting to detect malingering, albeit not foolproof [25].

Another electrophysical type of testing is the electro-olfactogram (EOG). An electrode is placed directly on the olfactory epithelium to measure the summated depolarization of olfactory receptors [46]. EOG has given valuable insight into the desensitization of repeated stimuli at short intervals [47]. However, similar to the use of EEGs, EOGs are not routinely used in clinical settings. The olfactory epithelium is difficult to access requiring the need for nasendosopy and precise placement of an electrode. Even with successful placement of the electrode, the rate of detecting the action potential is roughly 75% [48]. It is postulated that topological distribution of olfactory receptors is not uniform and has inter-individual variability, which makes consistent EOG readings challenging [49]. In addition, anaesthesia is not used during placement of electrodes as it may lower the amplitude of EOG and hence makes EOGs an unpleasant experience for the subject [48].

### Functional MRI

Functional MRI (fMRI) measures the changes in blood oxygen level and its flow that occur in response to an odour, typically using blood oxygen level-dependent (BOLD) contrast [50]. The biggest strength of fMRI is arguably its ability to elucidate functional neuroanatomy and map the key olfaction processors and subsequently use the information to assess presence of olfactory function [51]. Levy et al. demonstrated a significant quantitative reduction of brain activation in hyposmic patients in regions known to be important for olfactory processing and also detected changes in novel areas like the inferior frontal and cingulate gyral regions of the frontal cortex [52]. However, fMRI is still reserved for research purposes due to key challenges it has to overcome before it can become a routine part of clinical practice [53].

Similar to electrophysiological tests, fMRI pattern is sensitive to many parameters which ultimately presents as inter-individual variabilities of fMRI patterns to the same odour [51, 54]. New protocols with rigorous methodology are being developed to minimize inter-individual variabilities through short odour stimulation time, rapid odour repetition, and task protocols (passive odour exposure vs. active synchronised sniffing and breathing patterns) [55–57]. Another challenge for the routine use of fMRI in investigating olfactory disorders is the effect of subjective perceptions to the odour. Depending on familiarity and perceived pleasantness of the odour, fMRI patterns can differ [58]. Therefore, fMRI yet remains unreliable to detect generalizable group-level effects, but protocols and techniques should nevertheless continue to be optimised to allow more consistent detection of brain activity.

### Computerised Tomography (CT) Imaging

CT should be used to assess the paranasal sinuses for inflammatory disease [59, 60], and possibly with trauma and iatrogenic pathology, based on clinical suspicion from history and examination with nasendoscopy (e.g., where CSF rhinorrhoea is also present) [60]. The radiological assessment of the paranasal sinuses and ostiomeatal complex is routinely performed using the widely adopted Lund-Mackay score [61]. In this score, each sinus is assessed and assigned a score of either 0 (no abnormality), 1 (partial opacification), or 2 (complete opacification) with the ostiomeatal complex being assigned a score of either 0 (not obstructed) or 2 (obstructed). If on endoscopic examination or other imaging, an olfactory cleft stenosis is detected, then one can utilise CT imaging and volumetric techniques to evaluate the olfactory clefts of chronic rhinosinusitis subject. In this patient cohort, there is an inverse relationship between the percentage of olfactory cleft opacification and scores of psychophysical tests including SS and subjective olfactory-specific quality of life assessments [62, 63]. 

### Magnetic Resonance Imaging (MRI) Imaging

MRI has many uses in olfactory assessment. The use of a structural MRI can allow for the assessment of the olfactory apparatus, the exclusion of intracranial or sinonasal neoplasms, and the exclusion of asymptomatic chronic inflammation of the paranasal sinuses, can characterise traumatic brain injury (to assess cortical gliosis thus providing prognosis and treatment guidance), and can assess for neurodegenerative disorders [60, 64]. All the aforementioned have the potential to cause olfactory dysfunction [65•]. Furthermore, structural MRI can allow for the exclusion of other causes in patients that have apparent idiopathic olfactory loss [66, 67]. Olfactory bulb volumes and olfactory sulcus depth can be calculated using MRI, both of which are affected in numerous conditions including, congenital olfactory dysfunction (thus providing a prognosis for these patients), toxin exposure, and neurodegenerative diseases [68, 69]. Olfactory bulb volumes, once adjusted for age and gender, if shown to be hypoplastic or aplastic have been associated with reduced olfactory perception in many diseases [70]. There is often a vogue to request MRI and/or CT scans regardless of the suspected cause, but generally, there is no primary role for the use of MRI in patients suspected to have olfactory loss secondary to PIOD or iatrogenic causes [65•]. Moreover, MRI is not the radiological investigation modality of choice for patients with chronic rhinosinusitis—CT sinuses being preferred for this matter.

### Other Investigations

As described earlier, most olfactory disorders are secondary to conditions such as CRSwNP, traumatic head injury, viruses, drugs or toxins, and congenital anosmia [71]. Other medical conditions that are associated with olfactory dysfunction include endocrine disorders (Addison’s disease, Turner’s syndrome, or hypothyroidism), metabolic disorders (hypertension, vitamin B12 deficiency, diabetes mellitus), psychiatric conditions, migraines, alcohol dependence, syphilis, intranasal or intracranial neoplasms, or surgery [72–76]. Therefore, it is important to investigate these other causes when the history and examination indicate a suspected idiopathic case. A screening battery of blood tests that may elicit a cause include full blood count, urea and electrolytes (U&Es), liver function tests (LFTs); thyroid function tests (TFTs); angiotensin-converting enzyme (ACE); anti-neutrophil cytoplasmic antibodies (ANCA); zinc, iron, and magnesium levels; venereal disease research laboratory (VDRL); haemoglobin A1C (HbA1c); 9 am cortisol levels; and vitamin B12 and folate levels to screen for medical/metabolic causes (Table 1) [77].

**Table 1:** Screening blood tests for medical causes of olfactory disorders

**Screening blood test**	**Medical cause of olfactory disorder**
FBC	Anaemia
U&Es	Chronic renal dysfunction
LFTs	Hepatic dysfunction
TFTs	Hypothyroidism
ACE	Sarcoidosis
ANCA	Vasculitides
Zinc	Zinc deficiency
Iron	Iron deficiency anaemia
Magnesium	Hypomagnesemia
VDRL	Syphilis
HbA1c	Diabetes mellitus
9 am cortisol levels	Addison’s disease
Vitamin B12 and folate levels	Vitamin B12 and/or folate deficiency

### Outcomes for Olfactory Disorders

Clinical outcomes are important in both in clinical practice and in research as they demonstrate whether an intervention is effective on patients and trial participants and therefore can influence future clinical practice. It is important to have uniformity in the outcomes reported in research. This is to ensure that issues such as heterogeneity, selective reporting and bias and lack of relevance to clinical practice do not arise as a result of poor selection, measurement, or reporting of outcomes [78]. A core outcome set is an ‘agreed standardised collection of outcomes that should be measured and reported, as a minimum, in trials within a specific area’ [79]. It is expected that the core outcomes will always be reported as a minimum. If a COS is not used for a trial, there should be explanation for this. The COMET (core outcomes measurements in effectiveness trials) initiative is a collective group of researchers interested in the development of COS with a searchable database of COS [80]. To develop a COS involves identifying potential outcomes and ranking them to define a core set. It is important to involve stakeholders such as patients, clinicians, other healthcare professionals, and researchers to provide a complete picture of the COS from each group’s perspective. This is typically achieved using a multi-professional steering group [78].

A COS for olfactory disorders has been developed by the Clinical Olfactory Working Group (COWoG), a global panel of clinicians/researchers specialising in olfaction, who along with patient representatives were assembled and asked to rate a long list of outcomes covering domains including subjective questions, quality of life, rhinological, psychophysical, radiological, electrophysiological, pathophysiological, and acceptability of treatment and compliance. The list of outcomes for discussion was attained by a literature search. An iterative two-stage Delphi process is used to narrow the long list of outcomes to a final core outcome set [81]. This involves stakeholders scoring the importance of each outcome in each round. A summary was produced and sent back to each stakeholder for them to review and with an opportunity to change their own score with the knowledge of the overall group’s response. After the second round, the final list of COS for olfactory disorders was produced. A final list of COS for olfactory disorders includes visual analogue scores, quality of life measurements such as the Questionnaire of Olfactory Disorders (QOD) or EuroQol-5 Dimension (EQ-5D), psychophysical testing with SS or UPSIT, side effects related to medication, patient diary/symptom log, and baseline gustatory function assessment (Table 2). These COS are under review and pending publication [82]. This COS will increase the strength of future research including systematic reviews and meta-analyses. This will be reviewed in 5-year time to adapt for advancements in research.

**Table 2:** Finalised core outcome set for olfaction [82]

**Key COS** domains	**Choice of** outcome measures
Patient reported outcome measures	Quantitative and qualitative visual analogue score
Quality of life measures	QOD, EQ-5D
Psychophysical testing	SS/UPSIT
Presence of side effects (medication related) to the investigated medicinal product/device	Patient diary/symptom log
*Baseline gustatory function assessment (NOT an outcome measure)*	*Taste strips*

## Conclusions

A structured approach is required when assessing a patient presenting with olfactory dysfunction. A thorough history and nasal examination with endoscopy can reveal or exclude common aetiologies of olfactory dysfunction such as sinonasal inflammatory conditions, post-viral, or post-traumatic olfactory loss. It is well recognised that olfactory dysfunction can have an impact on a patient’s quality of life, and therefore, subjective assessment with validated questionnaires is important to assess this. The use of psychophysical testing is essential to measure olfactory function and guide prognosis and management with other investigation modalities being performed in the research setting. In apparent idiopathic cases, it is important to recognise other potential rare aetiologies with appropriate imaging and blood tests. The use of a core outcome set is essential in all future research in ODs to prevent heterogeneity between studies as well as reducing bias and ensuring clinical relevance.
